# Histamine modulates hippocampal inflammation and neurogenesis in adult mice

**DOI:** 10.1038/s41598-019-44816-w

**Published:** 2019-06-10

**Authors:** Cláudia Saraiva, Sandra Barata-Antunes, Tiago Santos, Elisabete Ferreiro, Ana Clara Cristóvão, Catarina Serra-Almeida, Raquel Ferreira, Liliana Bernardino

**Affiliations:** 1 CICS-UBI - Health Sciences Research Centre, University of Beira Interior, 6201-001 Covilhã, Portugal; 2 Center for Neuroscience and Cell Biology, 3004-504 Coimbra, Portugal

**Keywords:** Neurological disorders, Neuroimmunology, Adult neurogenesis

## Abstract

Evidence points to a dual role of histamine in microglia-mediated neuroinflammation, a key pathological feature of several neurodegenerative pathologies. Moreover, histamine has been suggested as a modulator of adult neurogenesis. Herein, we evaluated the effect of histamine in hippocampal neuroinflammation and neurogenesis under physiological and inflammatory contexts. For that purpose, mice were intraperitoneally challenged with lipopolysaccharide (LPS) followed by an intrahippocampal injection of histamine. We showed that histamine *per se* triggered glial reactivity and induced mild long-term impairments in neurogenesis, reducing immature neurons dendritic volume and complexity. Nevertheless, in mice exposed to LPS (2 mg/Kg), histamine was able to counteract LPS-induced glial activation and release of pro-inflammatory molecules as well as neurogenesis impairment. Moreover, histamine prevented LPS-induced loss of immature neurons complexity as well as LPS-induced loss of both CREB and PSD-95 proteins (essential for proper neuronal activity). Altogether, our results highlight histamine as a potential therapeutic agent to treat neurological conditions associated with hippocampal neuroinflammation and neurodegeneration.

## Introduction

Histamine is an endogenous biogenic amine classically associated with peripheral allergic and inflammatory reactions but it can regulate both brain inflammation^1^ and neurogenesis^2,3^. Histamine action is mediated by the activation of four different G protein-coupled receptors: histamine H1 receptor (H1R), H2R, H3R and H4R, which are differentially expressed in distinct brain cell phenotypes^4^. Several studies demonstrated the ability of histamine to modulate the brain inflammatory response by: (i) increasing microglial cell mobility through a signaling pathway involving α5β1 integrin, p-38 and Akt^5^; (ii) prompting the release of pro-inflammatory mediators, namely tumor necrosis factor alpha (TNF-α) and interleukin-6 (IL-6), through H1R and H4R^6^; (iii) promoting microglial phagocytic activity by the activation of H1R and (iv) inducing reactive oxygen species (ROS) production *via* Nox1 signaling pathway^7^. Thus, under a physiological context, histamine seems to induce a pro-inflammatory phenotype causing neuronal damage or even neuronal degeneration^7,8^. On the other hand, histamine has been shown to counteract LPS-induced inflammation. In fact, histamine decreased microglial migration, phagocytosis and ROS production induced by LPS as well as the release of interleukin-1β (IL-1β)^5^ and prostaglandin E2^9^. As such, histamine seems to have a dual role in the central nervous system (CNS) depending on the microenvironment, the activation state of cells and which histamine receptor is activated. Interestingly, it was recently demonstrated that initial peripheral inflammatory stimuli, such as LPS, generates immune memory in brain macrophages (microglia) resulting in a differential immune response to subsequent stimuli^10^. This immune memory contributes to the modulation of several neurological pathologies and might also explain the dual role of histamine.

Another important process regulated by histamine is adult neurogenesis, which occurs constitutively throughout life, mainly in the subventricular zone (SVZ) and in the hippocampal subgranular zone (SGZ). *In vitro* studies showed that H1R, H2R and H3R are expressed in neural stem cell (NSC) niches and that histamine induces NSC proliferation and neuronal differentiation through H2R and H1R signaling pathways, respectively^3,11–13^. In fact, histamine activation of H1R results in SVZ NSC differentiation *via* Mash1, DLX2 and Ngn1 into mature neurons, enabling the integration and differentiation of SVZ progenitors when grafted onto hippocampal slices or in the mouse hippocampus and striatum *in vivo*^3^. Moreover, intraventricular infusion of histamine into the lateral ventricles induces a significant increase in the number of SVZ neuroblasts capable of migrating towards the olfactory bulb where they differentiate into mature neurons^2^. Interestingly, H1R knockout mice present a reduced number of proliferative cells in the hippocampal dentate gyrus (DG) together with pronounced deficits in spatial learning and memory^14^. Additionally, the hippocampal inactivation of H3R, using the specific antagonist S38093, promoted neurogenesis in young and aged mice. Remarkably, the performance of aged mice in a context discrimination task was also improved^15^. These studies indicate that histamine might also modulate SGZ neurogenesis.

Altogether, unraveling the multiple actions of histamine might lead to the development of anti-inflammatory and regenerative therapies for both acute brain pathologies (e.g. ischemic stroke) and neurodegenerative disorders (e.g. Parkinson’s disease (PD), Alzheimer’s disease (AD)). Nevertheless, there is a lack of information regarding the effects of increased histamine levels in the hippocampus, a brain region that plays a key role in behavior and cognitive performance and that is compromised under neuroinflammatory conditions. In this sense, we evaluated the effects of histamine *per se* or under an inflammatory context mimicked by LPS in both hippocampal neuroinflammation and neurogenesis in adult mice. Herein, we report a pro-inflammatory role for histamine *per se* and its anti-inflammatory action after LPS stimulus. Moreover, we show that histamine can revert the loss of volume and arbor complexity of newborn neurons caused by LPS. Overall, we validate histamine as a potential therapeutic molecule in the fight against neuroinflammatory conditions.

## Materials and Methods

### Mice

All animal experiences were conducted in agreement with protocols approved by the national ethical requirements for animal research and the European Convention for the Protection of Vertebrate Animals Used for Experimental and Other Scientific Purposes (European Union Directive number 192 2010/63/EU). The *in vivo* experiments were carried out by investigators with the training required by present legislation (Functions B and C defined in article 23 of EU Directive 2010/63), and were authorized by the Animal-Welfare Body of the Health Research Centre at the University of Beira Interior (CICS-UBI) in compliance with the national legislation (EU Directive 2010/63, Chapter I, article 3). Two to five months-old C57BL/6J male mice were used. Mice were housed in the same room and in similar cages under controlled conditions: 12 h light/dark cycle at temperature of 22 °C and *ad libitum* access to food and water. All efforts were made to minimize the number of animals used in the study.

### Intraperitoneal and stereotaxic injections

Mice were initially subjected to an intraperitoneal injection (i.p.) of LPS (from Escherichia coli 055:B5, Sigma-Aldrich Co. LLC, St. Louis, MO, U.S.A.), at 1 mg/Kg^16^ or 2 mg/Kg^17^, diluted in 0.1 M sterile phosphate buffered-saline (PBS) at pH 7.4. Mice intraperitoneally injected with 0.1 M of sterile PBS were considered the control condition. Two days after LPS administration, mice were anesthetized with an i.p. injection of ketamine (90 mg/Kg of mouse weight; Imalgene 1000, Merial, Lyon, France) and xylazine (10 mg/Kg of mouse weight; Rompun 2%, Bayer, Leverkusen, Germany) and placed in a digital stereotaxic frame (51900 Stoelting, Dublin, Ireland). A unilateral intracerebral injection of 2 µL of sterile histamine dihydrochloride (100 μM His in PBS, Sigma-Aldrich Co. LLC; ipsilateral) was performed in the DG of the hippocampus (anteroposterior: -1.9 mm, mediolateral: -1.2 mm, and dorsoventral: -1.8 mm from bregma^3^) using a Hamilton syringe (Hamilton, Reno, NV, USA) at a speed of 0.2 µL/min. After intracerebral injection, the incision was sutured, and mice were kept warm (37 °C) until they recovered from anesthesia. Six experimental conditions were analyzed: i) contralateral hemisphere of mice subjected to i.p. injections of PBS and to intracerebral injection of 100 μM His - PBS contralateral group or “PBS”; ii) ipsilateral hemisphere of mice subjected to i.p. injections of PBS and to intracerebral injection of 100 μM His - “His”; iii) contralateral hemisphere of mice subjected to i.p. injections of 1 mg/Kg of LPS and to intracerebral injection of 100 μM His - “1 mg/Kg LPS”; iv) ipsilateral hemisphere of mice subjected to i.p. injections of 1 mg/Kg of LPS and to intracerebral injection of 100 μM His - “1 mg/Kg LPS + His”; v) contralateral hemisphere of mice subjected to i.p. injections of 2 mg/Kg of LPS and to intracerebral injection of 100 μM His - “2 mg/Kg LPS”; and vi) ipsilateral hemisphere of mice subjected to i.p. injections of 2 mg/Kg of LPS and intracerebral injection of 100 μM His - “2 mg/Kg LPS + His”. This categorization is only possible because no significant differences among the contralateral side of mice injected i.p. with saline and histamine in the hippocampus (ipsilateral side) were found when compared with both the ipsilateral and the contralateral sides of sham mice (i.p injection of saline followed by hippocampal injection of histamine, data not shown).

To unveil the effects of histamine in hippocampal neuroinflammation, animals were euthanized 2 days after histamine stereotaxic injection and brains were removed for immunoblot analysis. To label dividing cells and evaluate the effects of histamine in neuroblast proliferation in the DG, 5-bromo-2′-deoxyuridine (BrdU; 100 mg/Kg of animal weight, Sigma-Aldrich Co. LLC) dissolved in sterile saline solution (0.9% NaCl) was injected i.p. the following 2 days (every 12 h) after the stereotaxic procedure. Mice were maintained for 3 days after histamine treatment before being euthanized for further immunohistochemistry analysis. Lastly, to uncover the effects of histamine in the survival of newborn neurons and their dendritic complexity in the DG, BrdU i.p. injections (50 mg/Kg of animal weight in 0.9% NaCl) were performed every 12 h during the first 3 days after the histamine stereotaxic injection. Mice were euthanized six weeks after histamine treatment for further immunohistochemical analysis. Animal weight was daily monitored throughout all the experiments and no significant changes were observed (data not shown).

### Tissue collection

At day 2 after histamine stereotaxic injection, mice were decapitated and the hippocampi were dissected and immediately frozen in liquid nitrogen and stored at −80 °C until protein extraction processing and western blotting analysis.

At day 3 and week 6 after intracerebral injection, mice were deeply anesthetized with a mixture of ketamine and xylazine (90 mg/Kg and 10 mg/Kg of mouse weight, respectively) and perfused intracardially with saline solution, followed by 4% paraformaldehyde (PFA, Sigma-Aldrich Co. LLC). Brains were removed and post-fixed in 4% PFA for 24 h at 4 °C followed by immersion in a 30% sucrose solution (Fisher Scientific, Pittsburgh, PA, USA) until sunk. Then, brains were cryopreserved and 40 μm coronal sections of the hippocampus were collected in series of 12 sequential slices (spaced 460 µm each) using a cryostat-microtome (Leica CM3050S, Leica Microsystems, Nussloch, Germany). Slices were stored in cryopreservation solution (30% glycerol, 30% ethylene glycol and 10% phosphate buffer (0.2 M)) at −20 °C until immunohistochemistry staining.

### Western blotting

Hippocampal tissues were mechanically dissociated and lysed on ice in RIPA buffer (0.15 M NaCl, 0.05 M Tris, 5 mM ethylene glycol tetraacetic acid, 1% Triton X-100, 0.5% deoxycholic acid, 0.1% sodium dodecyl sulphate, 10 mM dichlorodiphenyltrichloroethane) containing a cocktail of proteinase inhibitors (Roche, Basel, Switzerland). The protein soluble fraction was obtained by centrifugation at 17,700 g for 20 min at 4 °C, after vortex homogenization. The total protein concentration from the lysates was determined using the bicinchoninic acid assay (BCA; Thermo Scientific, MA, USA). Protein samples were denatured in SDS-PAGE buffer (350 mM Tris, 10% (w/v) SDS, 30% (v/v) glycerol, 0.6 M DTT, 0.06% (w/v) bromophenol blue) for 5 min at 95 °C. Proteins (40 µg or 80 µg of total protein) were resolved in 8% or 12% SDS polyacrylamide gels at 90–100 V and then transferred to polyvinylidene difluoride (PVDF) membranes (GE Healthcare, Buckinghamshire, UK) in the following conditions (Trans-blot Turbo System, BioRad Laboratories, CA, USA): 1.0 A, 25 V, 15–30 min, using Towbin transfer buffer (25 mM Tris, 192 mM glycine pH 8.3, 20% methanol) at room temperature (RT). To block non-specific binding, the membranes were incubated with a tris-buffer saline (TBS) containing 0.1% Tween-20 (Thermo Fisher Scientific, Waltham, MA, USA), and 5% low-fat milk or 5% BSA (Amresco LLC, Solon, USA) or 0.1% gelatin (Fluka, St Louis, MO, USA), depending on the antibody used, for 20 min at RT. Membranes were then incubated overnight at 4 °C with appropriate primary antibodies diluted in blocking solution: mouse anti-ionized calcium binding adaptor molecule 1 (Iba-1; 1:200, Santa Cruz Biotechnology Dallas, TX, U.S.A.); mouse anti-glial fibrillary acidic protein (GFAP; 1:5000, Santa Cruz Biotechnology); rabbit anti-interleukin-1 beta (IL-1β; 1:200, HMGBiotech, Milano, Italy); mouse anti-high mobility group box 1 protein (HMGB1; 1:500, Cell Signaling, Beverly, MA, USA); rabbit anti-cAMP response element binding protein (CREB; 1:1000, Cell Signaling); mouse monoclonal anti-postsynaptic density protein 95 (PSD-95; 1:1000, Merck Millipore, Darmstadt, Germany). After washing with TBS-T, membranes were further incubated for 2 h at RT with the respective horseradish peroxidase-conjugated secondary antibody: goat anti-mouse, chicken anti-rabbit (1:5000; all from Santa Cruz Biotechnology) in blocking solution. To normalize the expression of the target proteins, the membranes were further incubated with a housekeeping antibody solution (90 min): mouse monoclonal anti-actin (1:1000, BD Biosciences; Franklin Lakes, NJ, U.S.A); mouse monoclonal anti-glyceraldehyde 3-phosphate dehydrogenase protein (GAPDH; 1:5000, Merck Millipore) and mouse anti-tubulin (1:5000, Sigma-Aldrich Co. LLC); followed by the respective secondary antibody (1 h), both at RT. Protein immunoreactive bands were visualized in a Chemidoc^TM^MP imaging system (BioRad Laboratories) after incubation with NZY supreme ECL reagent (NZYTech, Lisbon, Portugal). Densitometric analysis was performed using the software ImageLab (BioRad Laboratories). Full-length blots are included in the supplementary data.

### Immunohistochemistry

The immunostaining assays were performed using a protocol adapted from^18^. First, brain sections were incubated with 2 M HCl for 25 min at 37 °C to induce DNA denaturation. After washing with PBS, tissue sections were further incubated in a blocking solution containing 2% of horse serum (Life Technologies, Carlsbad, CA, USA) and 0.3% Triton X-100 (Fisher Scientific) diluted in 0.1 M PBS for 2 h at RT. After the blocking procedure, tissue sections were incubated for 72 h at 4 °C in the following primary antibodies (diluted in the blocking solution): rat monoclonal anti-BrdU (1:500, AbD Serotec, Raleigh, NC, USA), goat polyclonal anti-doublecortin (DCX; 1:500, Santa Cruz Biotechnology), or mouse monoclonal anti-NeuN (1:500, Merck Millipore). Then, sections were rinsed in PBS and incubated with Hoechst (1:1000; Sigma-Aldrich Co. LLC) and the respective secondary antibodies: Alexa Fluor-488 donkey anti-rat, Alexa Fluor-546 donkey anti-goat or anti-mouse (all 1:500; all Life Technologies), diluted in a solution containing 0.3% Triton X-100 in 0.1 M PBS, for 2 h at RT. Finally, sections were rinsed in PBS and mounted in Fluoroshield Mounting Medium (Abcam Plc., Cambridge, UK) for further analysis.

### Cell number and volume quantification

#### Neuroblast proliferation analysis

To assess neuroblast proliferation, fluorescence immunostaining z-stack projections of the DG were acquired in serial sections at 480 µm rostrocaudal intervals along the entire hippocampus (bregma −3.88 mm to bregma −0.94 mm) using an AxioObserver LSM 710 confocal microscope (Carl Zeiss, Jena, Germany) under a 40x oil immersion objective. BrdU-positive (BrdU^+^) and BrdU/DCX-double positive (BrdU^+^/DCX^+^) cells were counted in these serial sections using ImageJ software (NIH Image, Bethesda, MD, USA) in the SGZ of mice 3 days after histamine administration. Total number of BrdU^+^ and BrdU^+^/DCX^+^ cells was estimated using the Abercrombie formula: T = (*N* × *V*)/(*t* + *D*), in which T is the total number of cells, N is cell density, V is the total volume of the considered area, t is slice thickness (40 µm) and D is average cellular diameter (cell diameters from 6 random cells *per* experimental condition)^19^.

#### Survival of newborn neurons analysis

To assess survival of newborn neurons, BrdU^+^/NeuN^+^ cells were counted in serial sections at 240 µm rostrocaudal intervals along the entire hippocampus, using an AxioObserver LSM 710 confocal microscope under a 63x oil immersion objective. Total number of BrdU^+^/NeuN^+^ cells from the hippocampus of 6-week-old mice, after histamine treatment, was estimated by applying the Abercrombie formula, as described previously.

#### Area and volume quantification

To estimate area and volume, images of the DG were taken in serial sections at 240 or 480 μm rostrocaudal intervals along the entire hippocampus. The images were obtained using an AxioObserver LSM 710 confocal microscope under a 10x objective. The areas were estimated delineating a line around DG using the Fiji software (NIH, Bethesda, MD, USA). The volume was estimated through the equation: V (µm^3^) = ∑^n^_i=1_ A_i_ × d^19^, in which A is the area of each section and d corresponds to the interval between slices (240 or 480 μm).

### Dendritic morphology analysis

To assess neuroblast volume and dendritic complexity 3D reconstructions from confocal stack images of DCX^+^ cells obtained using an AxioObserver LSM 710 confocal under an 40x oil immersion objective were made. Mice euthanized 6 weeks after histamine treatment were used for this analysis. 3D reconstructions were made using the simple neurite tracer plugin in Fiji Software^20,21^. For this purpose, we analyzed 20 DCX^+^ cells in “1 mg/Kg LPS” and “1 mg/Kg LPS + His” conditions and 30 DCX^+^ cells for “PBS”, “His”, “2 mg/Kg LPS” and “2 mg/Kg LPS + His” conditions from 3 sequential coronal sections separated by 240 µm of the suprapyramidal region of the SGZ (bregma −2.3 mm to bregma −1.8 mm) from 2 or 3 different mice, respectively. Dendritic morphology was analyzed in the Fiji software using the 3D Sholl analysis plugin that quantifies the number of intersections between Sholl circles (with a 15 µm increment) and dendrites. After the 3D reconstruction of the entire neuronal dendritic tree, the individual neuronal volume was also quantified using DCX staining and the fill out function in the Simple neurite tracer plugin^21^.

### Data analysis

Data are shown as the mean ± standard error of the mean (SEM), expressed as either percentages of values obtained in PBS condition or absolute values. Statistical analysis was performed using one-way ANOVA, followed by the post-hoc test Dunnett Multiple Comparison Test to measure differences between the PBS condition and the other experimental conditions. To assess differences between the pairs LPS condition and LPS + His condition for either concentration of LPS (1 or 2 mg/Kg) the one-way ANOVA followed by the Sidak Multiple Comparison Test was used. For Sholl analysis a two-way ANOVA, followed by Tukey test was used. In this way, for every distance from the soma assessed, the mean of the intersection numbers obtained for each condition were compared with all the other conditions. F values are described for each ANOVA analysis and values of P < 0.05 were considered significant. All statistical analysis was done using GraphPad Prism 8 (GraphPad Software, San Diego, CA, USA).

## Results

### Histamine modulates inflammation in mouse hippocampus

**Figure 1:**
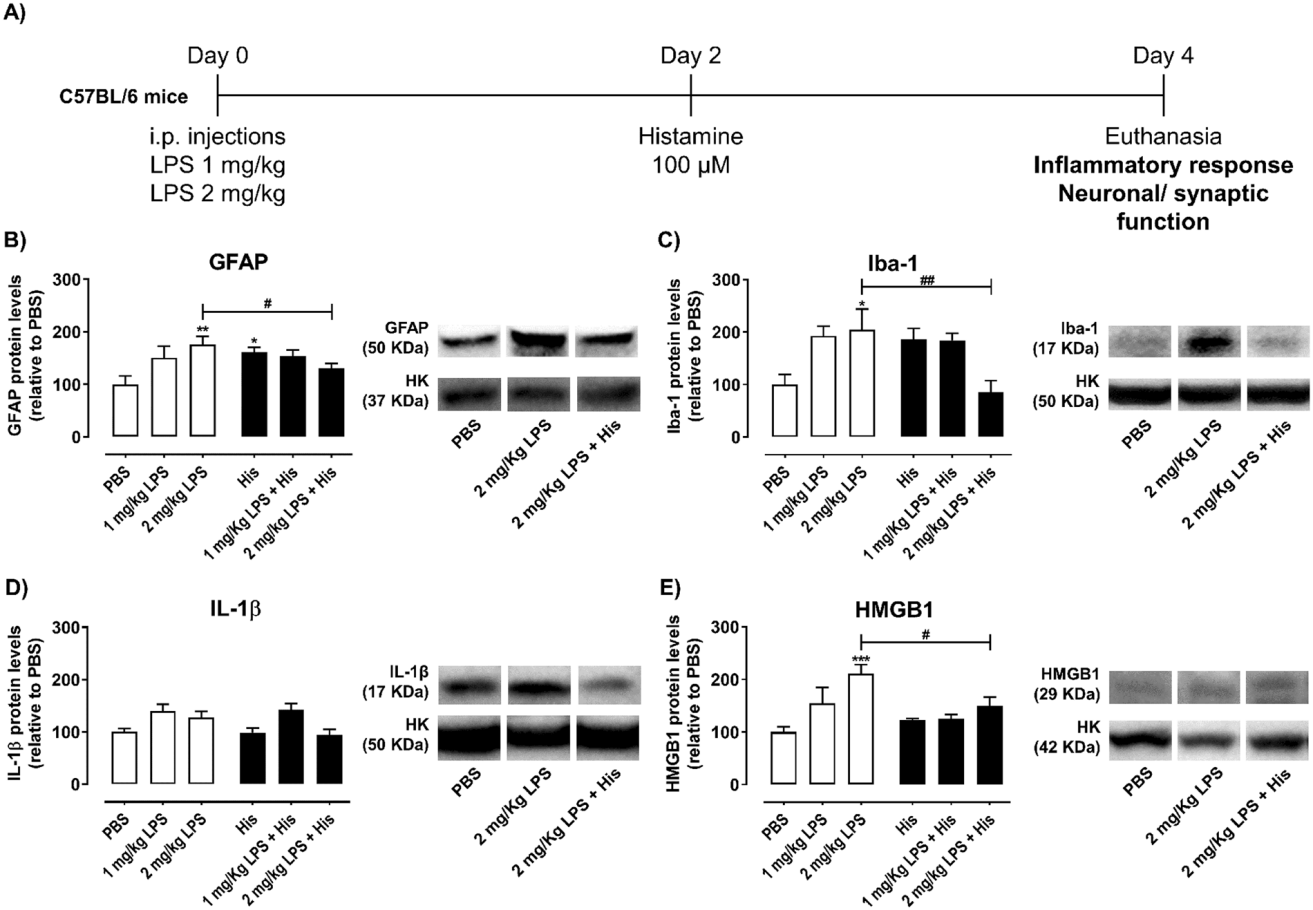
Histamine inhibits LPS-induced glial reactivity in the mouse hippocampus. (**A**) Adult mice were subjected to intraperitoneal injections (i.p.) of either PBS or LPS (day 0), followed by intracerebral administration of histamine in the hippocampus (day 2). Protein extraction from the hippocampus was performed at day 4. Bar graphs depict the percentage relative to PBS condition of GFAP (**B**), Iba-1 (**C**), IL-1β (**D**) and HMGB1 (**E**) protein expression. White bars represent mice contralateral hemisphere and black bars the ipsilateral hemisphere. On the right of each bar graph, representative image of the respective protein analyzed and the housekeeping (HK) protein used (GAPDH, 37 KDa; tubulin, 50 KDa; and actin, 42 KDa) are shown. Data are expressed as percentage of PBS ± SEM. Statistical analysis was performed using one-way ANOVA, followed by Dunnett Multiple Comparison Test (n = 4–7; *P < 0.05, **P < 0.01 and ***P < 0.001 when compared with PBS condition). Comparison of the following pairs: 1 mg/kg LPS vs 1 mg/kg LPS + His and 2 mg/kg LPS vs 2 mg/kg LPS + His was done by one-way ANOVA followed by the Sidak Multiple Comparison Test (^#^P < 0.05, ^##^P < 0.01).

First, to identify the role of histamine in hippocampal inflammation we assessed by western blotting the levels of GFAP and Iba-1 proteins to correlate with the levels of activated astrocytes and microglia, respectively, as well as the expression of the inflammatory mediators IL-1β and HMGB1 in LPS-challenged mice treated or not with histamine (Fig. 1). We observed that histamine *per se* significantly increased the reactivity of astrocytes (GFAP: PBS 100.0 ± 15.9, His 161.7 ± 8.8, n = 5–6, F = 4.016, P = 0.0126; Fig. 1B) and tended to increase the reactivity of microglia (Iba-1: PBS 100.0 ± 15.9, His 186.7 ± 20.3, n = 7, F = 4.226, P = 0.0716; Fig. 1C). As expected, both LPS dosages induced glial reactivity with 2 mg/Kg promoting a higher inflammatory response. The glial reactivity induced by 2 mg/Kg of LPS was reversed by the local administration of 100 µM of histamine (GFAP: 2 mg/Kg LPS, 176.0 ± 15.6; 2 mg/Kg LPS + His, 130.8 ± 8.9, n = 6–7; P = 0.0147; Iba-1: 2 mg/Kg LPS, 204.4 ± 39.6; 2 mg/Kg LPS + His, 85.6 ± 22.1, n = 7; P = 0.0017; Fig. 1B,C). Not surprisingly, LPS-challenged mice had higher hippocampal levels of the TLR4-mediated pro-inflammatory mediators IL-1β and HMGB1 (Fig. 1D,E), with histamine being able to counteract LPS-induced HMGB1 increased expression when 2 mg/Kg of LPS was used (PBS, 100.0 ± 9.9; 2 mg/Kg LPS, 211.1 ± 17.2; 2 mg/Kg LPS + His, 129.9 ± 2.6, n = 3–4; F = 6.438, ***P = 0.0004, ^#^P = 0.0109). Although not statistically significant, a similar effect was observed for IL-1β.

### Histamine modulates mouse hippocampal neurogenesis

**Figure 2:**
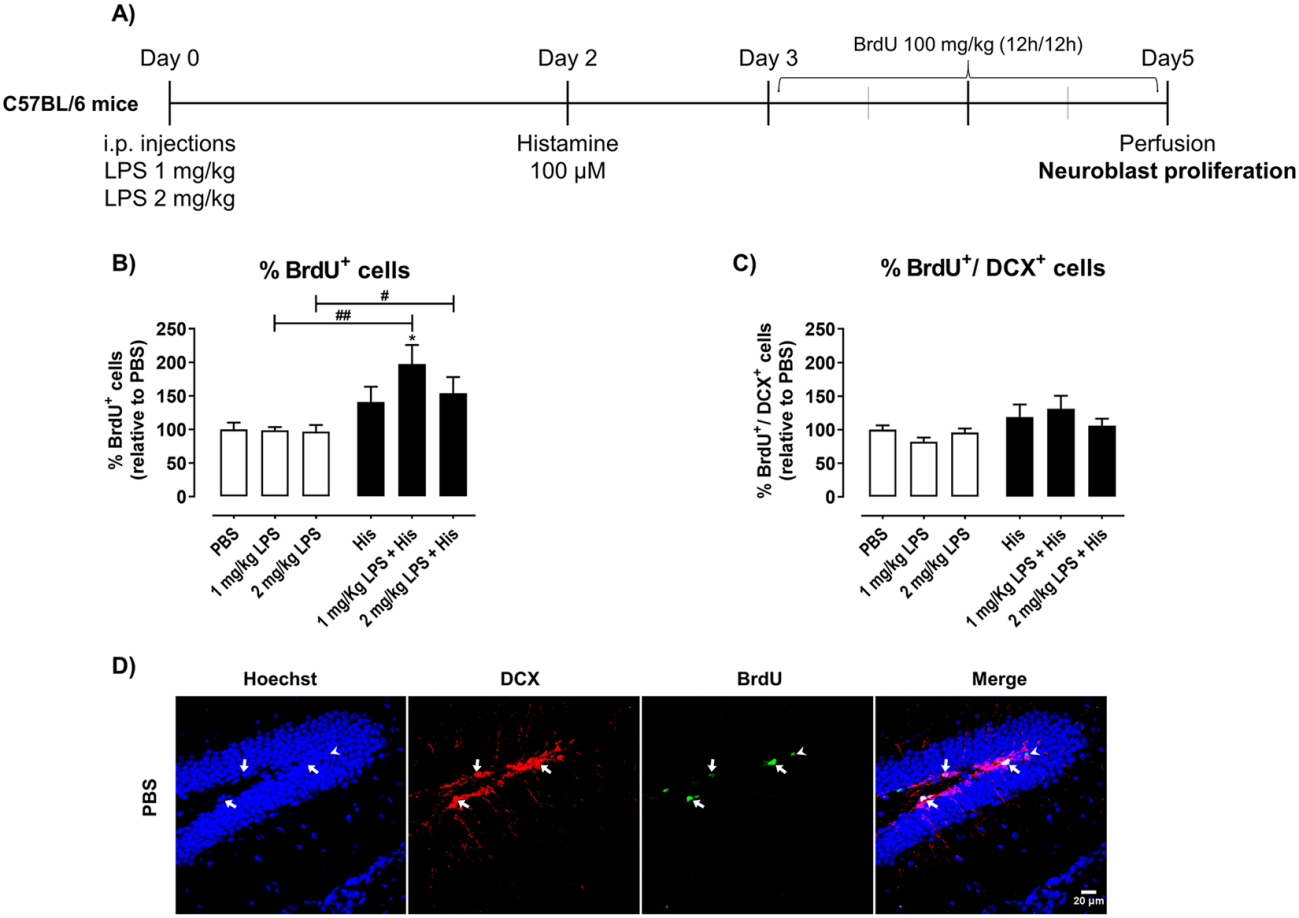
Histamine promotes cell proliferation in hippocampal DG. (**A**) Adult mice were subjected to intraperitoneal injections (i.p.) of either PBS or LPS (day 0), followed by intracerebral administration of histamine in the hippocampus (day 2). Then, mice received intraperitoneal injections of 100 mg/Kg of BrdU every 12 h (day 3 and 4). Thereafter, mice were perfused for histological analysis at day 5. Graphs depict the percentage relative to PBS experimental condition of total number of proliferative BrdU^+^ cells (**B**) and of BrdU^+^/DCX^+^ cells (**C**) in the hippocampal DG. **D**) Representative confocal images of BrdU (green; white head arrow) and DCX (red; white arrows) were obtained in hippocampal DG mouse slices. Nuclear staining is in blue. Scale bar is 20 μm. The data are expressed as percentage of PBS ± SEM. Statistical analysis was performed using one-way ANOVA, followed by Dunnett Multiple Comparison Test (n = 3–6; *P < 0.05 when compared to control condition). Comparison of the following pairs: 1 mg/kg LPS vs 1 mg/kg LPS + His and 2 mg/kg LPS vs 2 mg/kg LPS + His was done by one-way ANOVA followed by the Sidak Multiple Comparison Test (^#^P < 0.05, ^##^P < 0.01).

Neuroinflammation has been implicated in the impairment of adult neurogenesis^16^. Consequently, to unveil the effects of histamine in SGZ neurogenesis, we assessed the total number of proliferative cells (BrdU^+^ cells) as well as the levels of dividing neuroblasts (BrdU^+^/DCX^+^ cells) along the entire SGZ, in mice exposed to saline or LPS (1 or 2 mg/Kg) and later on treated with 100 µM of histamine (Fig. 2A). We found that histamine positively influenced the levels of total BrdU^+^ cells without altering the levels of BrdU^+^/DCX^+^ cells when compared with control. Nevertheless, in mice exposed to LPS, histamine significantly increased the total amount of proliferative cells (PBS, 100.0 ± 10.1; 1 mg/Kg, LPS 98.8 ± 4.7; 1 mg/Kg of LPS + His, 197.6 ± 28.2, 2 mg/Kg, LPS 96.7 ± 9.7; 2 mg/Kg of LPS + His, 154.1 ± 23.8 n = 3–4; F = 3.579, *P = 0.0326, ^##^P = 0.081, ^#^P = 0.0251; Fig. 2B), but did not change the levels of BrdU^+^/DCX^+^ cells (Fig. 2C). Interestingly, LPS did not show an effect in DG overall proliferation nor in the number of proliferative neuroblasts (Fig. 2B,C).

**Figure 3:**
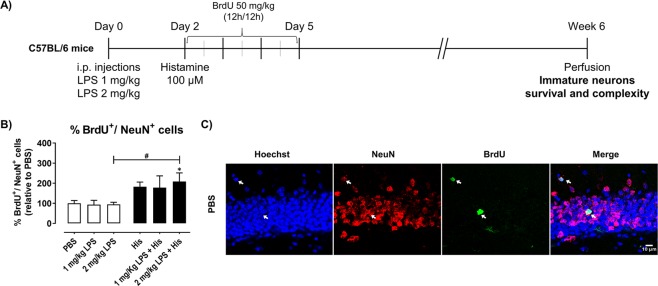
Histamine enhances survival of newborn neurons in the hippocampal DG in LPS-challenged mice. Adult mice were subjected to intraperitoneal injections (i.p.) of either PBS or LPS (day 0), followed by intracerebral administration of histamine in the hippocampus (day 2). Then, mice received intraperitoneal injections of 50 mg/Kg of BrdU every 12 h (day 3, 4 and 5). Thereafter, mice were perfused for histological analysis at the end of week 6. (**B**) Graph represents the percentage relative to PBS of total number of BrdU^+^/NeuN^+^ neurons in the hippocampal DG. (**C**) Representative confocal images of cell nuclei (Hoechst, blue), BrdU (green; white arrows) and NeuN (red; white arrows) were obtained in hippocampal DG mouse slices. Scale bar is 10 μm. Data are expressed as percentage of PBS ± SEM. PBS condition was set to 100%. Statistical analysis was performed using one-way ANOVA followed by Dunnett Multiple Comparison Test (^*^P < 0.05 compared to PBS condition). One-way ANOVA followed by the Sidak Multiple Comparison Test was used to assess differences between 2 mg/kg LPS and 2 mg/kg LPS + His, ^#^P < 0.05.

We have further evaluated the effects of histamine *per se* and under LPS challenge in the long-term survival of newborn neurons (BrdU^+^/NeuN^+^ cells) in mice exposed to saline or LPS (1 or 2 mg/Kg) and treated with 100 µM of histamine 2 days later, which were euthanized 6 weeks after histamine administration (Fig. 3A). Histamine *per se* tended to increase the number of BrdU^+^/NeuN^+^ cells (Fig. 3B,C), while no significant effect was induced by any of LPS concentrations tested. Notably, histamine in the presence of a pre-conditioning LPS stimulus significantly increased the number of BrdU^+^/NeuN^+^ cells (PBS, 100.0 ± 13.6; 1 mg/Kg LPS, 93.5 ± 21.2; 1 mg/Kg LPS + His, 178.3 ± 58.3; 2 mg/Kg LPS, 94.6 ± 10.4; 2 mg/Kg LPS + His, 208.7 ± 42.8, n = 2–4; F = 3.568, *P = 0.0477, ^#^P = 0.0130 Fig. 3B).

### Histamine ameliorates the loss of neuronal complexity of hippocampal neuroblasts caused by LPS

**Figure 4:**
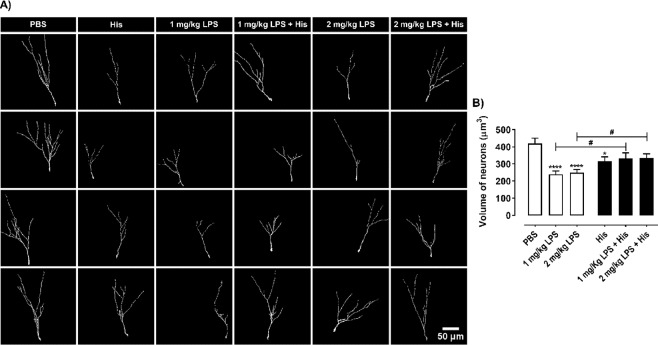
Histamine counteracts volume loss of hippocampal DG immature neurons caused by LPS. Adult mice were subjected to intraperitoneal injections of either PBS or LPS (day 0), followed by intracerebral administration of histamine in the hippocampus (day 2). Thereafter, mice were perfused for histological analysis at the end of week 6. (**A**) Three dimensional reconstructions of representative DCX^+^ cells of the hippocampal DG. Scale bar 50 μm. (**B**) Graph represents the volume of the dendritic tree from the reconstructed DCX^+^ immature neurons. Statistical analysis was performed using one-way ANOVA, followed by Dunnett Multiple Comparison Test (n = 20–30 cells from at least 2 different animals; *P < 0.05 and ****P < 0.0001 when compared to control condition). Comparison of the following pairs: 1 mg/kg LPS vs 1 mg/kg LPS + His and 2 mg/kg LPS vs 2 mg/kg LPS + His was done by one-way ANOVA followed by the Sidak Multiple Comparison Test, ^#^P < 0.05.

**Figure 5:**
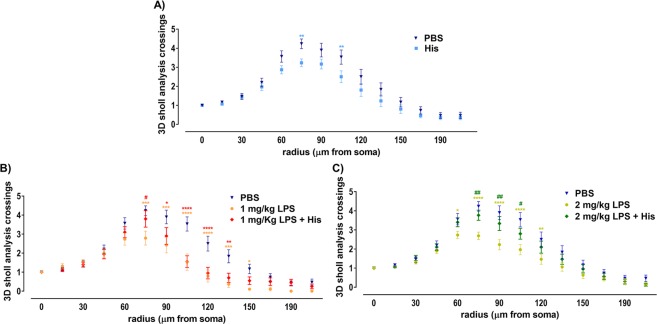
Histamine enhances dendritic complexity of hippocampal DG neuroblasts in LPS-challenged mice. Adult mice were subjected to intraperitoneal injections of either saline or LPS (day 0), followed by intracerebral administration of histamine in the hippocampus (day 2). Thereafter, mice were perfused for histological analysis at the end of week 6. (**A**–**C**) 3D Sholl analysis of DCX^+^ cells from the hippocampal DG. Graphs represent the number of dendritic intersections with Sholl circles in each radius comparing: PBS condition and histamine condition (**A**); PBS, 1 mg/Kg LPS and 1 mg/Kg LPS + Histamine conditions (**B**); and control, 2 mg/Kg LPS and 2 mg/Kg LPS + Histamine conditions (**C**). Statistical analysis was performed using two-way ANOVA, followed by the Tukey’s Comparison Test (n = 20–30 cells from at least 2 different animals; *P < 0.05, **P < 0.01, ***P < 0.001 and ****P < 0.0001 when compared to PBS condition; ^#^P < 0.05 and ^##^P < 0.01 when compared to the respective LPS-treated condition).

To further understand the long-term effects of histamine *per se* or under inflammatory conditions in the population of newborn neurons, we assessed the size and the dendritic complexity of immature neurons 6 weeks after histamine treatment (Fig. 3A). DCX^+^ cells from these animals were 3D reconstructed and the volumetric integration of DCX^+^ cells (Fig. 4) and DCX dendritic complexity (Fig. 5) evaluated. We observed that histamine *per se* and both concentrations of LPS induced a significant volumetric reduction of the dendritic arborization of DCX^+^ cells. On the other hand, in the presence of the inflammatory pre-conditioning stimulus (LPS), histamine was able to revert the LPS-induced loss of neuronal volume (PBS, 418.6 ± 32.7; His, 316.4 ± 24.6; 1 mg/Kg LPS, 238.6 ± 19.7; 1 mg/Kg LPS + His, 331.1 ± 33.4; 2 mg/Kg LPS, 248.7 ± 18.8; 2 mg/Kg LPS + His, 334.3 ± 24.8, n = 20–30 cells of at least 2 different animals, F = 6.20, *P = 0.0194, ****P < 0.0001; ^#^P = 0.0363, ^#^P = 0.0164, respectively; Fig. 4). The number of dendritic intersections with Sholl circles was also analyzed to measure dendritic complexity using ordinary two-way ANOVA analysis followed by Tukey’s Multiple Comparison Test, F = 69.96, P < 0.0001. Histamine alone led to a reduced number of crossings with the Sholl circles with a 65–85 µm and 105 µm radius compared with control mice (Fig. 5A). As expected, LPS also induced a reduction in dendritic complexity (Fig. 5B,C). At the concentration of 1 mg/Kg of LPS the number of dendritic intersections with a 65–140 µm Sholl radius was significantly reduced (Fig. 5B), while at the concentration of 2 mg/Kg this reduction was observed between the 60–130 µm Sholl radius. In agreement with previous results, histamine treatment of LPS-challenged mice reverted to some extent the LPS-induced loss of dendritic complexity. In 1 mg/Kg LPS-treated mice, histamine increased significantly the number of dendritic intersections at the Sholl radius 75 µm (Fig. 5B). This positive effect in the neuronal complexity was more evident when mice previously received 2 mg/Kg of LPS, since histamine increased significantly the number of dendritic crossings in the Sholl radius 70–105 µm (Fig. 5C).

### Histamine reverts synaptic plasticity loss caused by LPS in the mouse hippocampus

**Figure 6:**
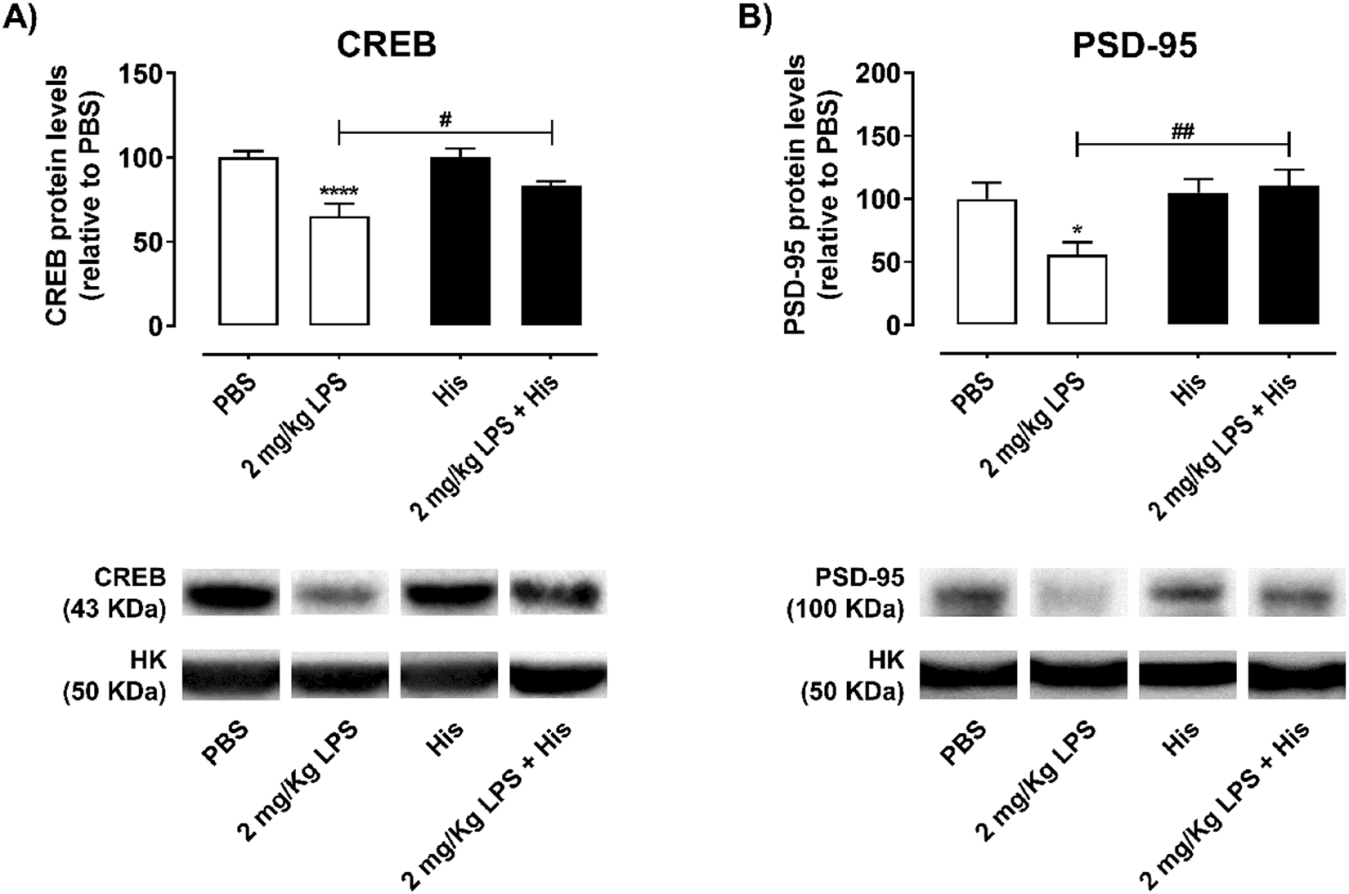
Histamine improves neuronal functionality in the hippocampus of LPS-challenged mice. Adult mice were subjected to intraperitoneal injections of either PBS or LPS (day 0), followed by intracerebral administration of histamine in the hippocampus (day 2). Protein extraction from the mice hippocampus was performed at day 3. Graphs depict the percentage relative to PBS of (**A**) CREB (**B**) PSD-95 protein expression in hippocampal samples. Bellow the graphic representative images of the western blotting analysis for CREB, PSD-95 and the housekeeping (HK) protein (tubulin, 50 KDa) are shown. Data are expressed as percentage of PBS ± SEM. Statistical analysis was performed using one-way ANOVA, followed by Dunnett Multiple Comparison Test (n = 3–7; *P < 0.05 and ****P < 0.0001 when compared to control condition). Comparison among 2 mg/Kg LPS condition and the 2 mg/Kg LPS + His condition was done by the Sidak Multiple Comparison Test after performing one-way ANOVA analysis (^#^P < 0.05 and ^##^P < 0.01).

Finally, to understand if the loss of dendritic complexity observed in 2 mg/Kg LPS-challenged mice and if the protective role of histamine was preceded by any alterations in terms of synaptic activity or neuronal function, the total levels of PSD-95 and CREB were evaluated by western blotting. The hippocampal tissue was obtained from mice exposed to the experimental setup described in Fig. 1A. CREB is a nuclear transcription factor that modulates neuronal plasticity and cognition^22^. CREB downregulation and signaling dysfunction have been implicated in neuroinflammatory conditions^23^. As such, evaluation of both CREB and the postsynaptic protein PSD-95 can be used as indirect indicators of neuronal functionality. Herein, we observed a significant reduction in the levels of both CREB and PSD-95 in the 2 mg/Kg of LPS condition. Histamine *per se* did not alter CREB and PSD-95 levels (Fig. 6A,B). On the other hand, when mice were pre-conditioned with LPS, histamine prevented the reduction of CREB and PSD-95 protein levels (CREB: PBS, 100.0 ± 3.9; 2 mg/Kg LPS, 65.3 ± 7.2; 2 mg/Kg LPS + His, 83.1 ± 2.7; n = 6–7, F = 11.15, ****P < 0.0001; PSD-95: PBS, 100 ± 12.7; 2 mg/Kg LPS, 55.8 ± 9.9; 2 mg/Kg LPS + His, 110.1 ± 12.9; n = 6, F = 4.59, *P = 0.0370, ^##^P = 0.0036; Fig. 6A,B).

## Discussion

Evidence has been pointing to neuroinflammation as a trigger in neurodegenerative disorders and cognitive decline^24–26^. In the brain, histamine can act either as neurotransmitter or as modulator of the innate immune system. Herein, we investigated the effects of histamine in mice hippocampal inflammation and neurogenesis, both in physiological conditions and under peripheral inflammation. For that purpose, mice were subjected to i.p. administration of LPS at two different dosages followed by the intrahippocampal administration of histamine.

Concomitant with other studies^5,7,8,27^, we also observed that in physiological conditions histamine acts as a pro-inflammatory mediator, increasing both astrocytic and microglia reactivity. Nevertheless, no alterations in the levels of IL-1β or HMGB-1, two pro-inflammatory molecules, were observed. The maintenance of IL-1β levels after histamine stimulus was also previously demonstrated by us on a microglial cell line (N9) and in hippocampal organotypic slice cultures^5^. On the other hand, the peripheral administration of LPS increased both astrocytic and microglia reactivity as well as production of pro-inflammatory molecules. Interestingly, histamine was able to inhibit glial reactivity and the release of pro-inflammatory molecules in mice previously challenged with 2 mg/Kg of LPS. Noteworthy, we have previously showed that co-administration of histamine with LPS does not induce cell death in a microglial cell line^5^. Moreover, no alterations in cleaved caspase-3 protein levels (an indicator of apoptosis) were observed in this study (data not shown), indicating that glial reactivity reduction does not derive from microglial cell death. Our data are in agreement with previous studies showing that histamine can have a dual role in the modulation of inflammation depending on the microenvironment and the activation state of glial cells^5,9,28^.

LPS administration is one of the most used and well-characterized approaches to induce inflammation, both in the CNS and periphery. LPS signals mainly through toll-like receptor 4 (TLR-4), which is located on microglia, astrocytes and endothelial cells in the CNS^29^. Some studies have reported that LPS induces an upregulation of the expression of histamine receptors. A higher H1R expression was reported in human coronary artery endothelial cells (HCAEC) after LPS exposure in a dose-dependent manner^30^. In a rat microglial cell line, stimulation with LPS (100 ng/mL) also resulted in a higher expression of the H4R (peak 24 h). Similarly, significant increase of H4R expression levels (from day 1 to at least day 7) were observed in the cortex of rats subjected to intracerebroventricular injections of LPS (1 µg/µl)^31^. Interestingly, a recent study showed that a single peripheral administration of different dosages of LPS (100 µg/Kg or 1 mg/Kg) resulted in a differential inflammatory response in the brain^32^. In fact, the authors reported that low dosages of LPS (100 µg/Kg) promote microglial activation only in the circumventricular organs and neighboring regions, while the higher concentration of LPS (1 mg/Kg) was able to activate microglia in many other regions (e.g. DG, corpus callosum, cerebellum, etc). Nevertheless, it is important to notice that even microglia reactivity in the DG after administration of 1 mg/Kg of LPS was still low comparing with other brain regions^32^, indicating that higher concentrations of LPS might be necessary to lead to a more robust inflammatory response in this region. Moreover, there are recent evidence of an innate brain immune memory caused by peripheral stimuli. Wendeln and colleagues recently demonstrated that inflammatory peripheral stimuli, such as LPS, induce an immune memory in the brain. This effect was mainly mediated by microglia, leading to either training (enhancing immune response) or tolerance (suppressing immune response). Interestingly, the immune memory, which occurs through epigenetic reprogramming, was also able to affect the severity of subsequent neurological disorders for at least 6 months in mice^10^. Inflammatory memory in epithelial stem cells was also similarly described, leading to wound healing promotion^33^.

Herein, we demonstrate the histamine potential to counteract LPS-induce damage in the hippocampus of mice that is more pronounced when a higher concentration of LPS (2 mg/Kg) is given. Considering the previously mentioned studies and our results, the superior anti-inflammatory and pro-neurogenic response induced by histamine when 2 mg/Kg LPS was given might be due to a weaker inflammatory response due to low availability of LPS in the DG when 1 mg/Kg is used and/or the acquisition of immune tolerance when 2 mg/Kg of LPS is administered. Nevertheless, the anti-inflammatory role induced by histamine reported by us is in agreement with other reports showing that histamine is able to suppress LPS-induced inflammation in human monocytes^34,35^, human monocyte-derived dendritic cells^36^, in microglia^1,9^, in the liver, and in the substantia nigra^1,37,38^. In these situations, histamine reduced the expression of pro-inflammatory cytokines (e.g. TNF-α, INF-γ, IL-18), led to cytoskeleton rearrangements and inhibited microglial activation, namely in terms of the phagocytic activity and ROS production^1,9,36–38^. Since LPS induces an upregulation of histamine receptors, a higher concentration of LPS (2 mg/kg) may be needed to boost its anti-inflammatory and protective effects in the DG.

Herein, we did not evaluate the mechanisms underlying the anti-inflammatory effects driven by histamine under LPS treatment. We have showed previously that the protective effect of histamine on a microglia cell line (N9) and hippocampal organotypic slices under LPS challenge was mediated by H4R^5^. Later we also demonstrated that histamine decreases microglial phagocytosis activity and ROS production induced by LPS in both the N9 cell line and in mouse primary microglia cultures. This effect of histamine was shown to be at least partially modulated by H1R and H4R. In contrast, the dopaminergic cell death induced by LPS in the mouse *substantia nigra* was rescued by histamine exposure through H1R^7^. Iida and collaborators showed that in mouse primary microglia cultures the H3R (presynaptic receptor that inhibits synthesis and release of histamine and other neurotransmitters) agonist imetit inhibited microglial chemotaxis, phagocytosis and LPS-induced production of cytokines^9^. The same authors have later on shown the opposite results in *ex vivo* mouse hippocampal organotypic slices and in *in vivo* conditions, since H3R inverse agonist (JNJ10181457) led to a reduction in LPS-induced microglial phagocytosis and cytokine expression as well as to the attenuation of depression-like behaviors caused by LPS^39^. Interestingly, Guilloux and collaborators recently showed that chronic administration of the H3R antagonist/reverse agonist S 38093 in mice promotes hippocampal neurogenesis in young adult mice, aged mice and in a transgenic model of AD. Moreover, this treatment was able to improve the performance of aged mice in a context discrimination test^15^. These studies demonstrate that the histamine mode of action might vary depending on the experimental conditions (e.g. *in vitro*, *in vivo* studies; brain regions; amount of histamine administered; type of pathology/stimuli used). Therefore, further studies are needed to disclose the type of histamine receptors and signaling pathways involved in the histamine protective role in neurological disorders with an inflammatory component (e.g. ischemic stroke, AD, PD, multiple sclerosis)^1,40^.

Inflammation has a negative effect on adult neurogenesis leading to abnormal cognitive and behavioral performance^16,41^. Importantly, we have previously shown that histamine promotes neurogenesis in the SVZ-olfactory bulb axis of adult healthy mice^2^. Moreover, chronic treatment of mice with H3R antagonist resulted in adult hippocampal neurogenesis potentiation and a decrease of cognitive deficits^15^, indicating that histamine can be used as a potential therapeutic molecule against neurological disorders. Herein, we unveiled the effects of histamine in adult hippocampal neurogenesis in physiological conditions and under systemic inflammation induced by LPS. In physiological conditions histamine *per se* slightly improved SGZ cell proliferation (BrdU^+^ cells). Nevertheless, at this time point it causes mild impairments in terms of immature neuron dendritic volume and complexity. The SGZ response to histamine was different from the SVZ one previously reported by us^2,3^. These two neurogenic niches present different neural/progenitor stem cell populations that may be differentially responsive to histamine. Possibly, SGZ and SVZ express different histaminergic receptors. On the other hand, LPS (independently of the concentration used) did not change SGZ neuroblast proliferation (BrdU^+^/DCX^+^ cells) nor neuroblast/immature neuron survival (as suggested by the unaltered number of BrdU^+^/NeuN^+^ cells). Nevertheless, it did significantly affect immature neuron dendritic volume and ramification number. In fact, others have shown that peripheral LPS impairs DG NSC survival without affecting their differentiation fate^42^. NSC cell cycle in the DG of adult mice is about 14 hours^43^. As such, the total number of BrdU^+^ cells evaluated in the SGZ did not only include proliferative NSC but also differentiated neural cell lineages (neuroblasts and astrocytes) or microglia. LPS has been reported to increase the proliferation of astrocytes and microglia^44^, making the evaluation of BrdU^+^/DCX^−^ cells relevant to better understand how proliferation occurs in the SGZ niche under the influence of LPS. Moreover, Valero and colleagues did not observe changes in the survival of newborn neurons after 7 weeks of LPS administration but detected a significant decrease in the number and volume of newborn neurons (DCX^+^ cells) originated long after the LPS challenge. Furthermore, LPS impaired the formation of synaptic specializations in the dendrites of DCX^+^ cells and induced long-lasting memory deficits^16^.

Interestingly, histamine treatment after LPS stimuli tend to promote the overall proliferation of SGZ and increase SGZ neuronal survival (BrdU^+^/NeuN^+^ cells). Moreover, histamine was able to significantly revert the loss of neuronal volume and arbor complexity caused by LPS. This effect was more pronounced for mice challenged by 2 mg/Kg LPS. These data are in agreement with what we observed for glial reactivity. Histamine modulatory effect on adult hippocampal neurogenesis seems to be influenced by the presence of a pre-conditioning peripheral inflammatory stimulus and in specific parameter associated with neurogenesis evaluation, by the stimuli intensity. This differential response might be associated with distinct immunological responses observed for each LPS stimulus. On the other hand, death of newborn cells in the mice DG occurs preferentially between 24 hours and 4 weeks in mice^45^, which may indicate an enhancement of cell survival during this period induced by histamine. Moreover, the results are also in accordance with previous reports showing a profound detrimental long-term effect of LPS on adult neurogenesis^16,46^.

Herein we showed that mice challenged with 2 mg/Kg of LPS had a more pronounced inflammatory response and long-term deficits in neurogenesis. For this LPS concentration, an early (short-term) alteration of both neuronal function (CREB levels) and synaptic plasticity (PSD-95 levels) was observed, while no changes were observed in mice only treated with histamine. CREB is a transcription factor involved in cognition and neuronal excitability^47^, and can be used as an indirect marker of neuronal functionality. PSD-95, the most abundant scaffold protein in the postsynaptic density, is a powerful regulator of synaptic strength^48^. Similarly, others reported downregulation of CREB activation in the hippocampus and prefrontal cortex of adult mice after peripheral LPS administration^49^. Although the total amount of CREB does not necessarily reflect its transcriptional sensitivity (phosphorylation required for CREB activity), decrease of the total CREB levels in LPS-challenged mice is indicative of hippocampal dysfunction. In fact, lower mRNA and protein levels of this molecule were observed in the hippocampus of AD mouse models and patients^50,51^. Likewise, decreased levels of PSD-95 have been found in aged individuals with dementia and AD patients^52,53^, indicating a correlation between PSD-95 levels and cognition decline. Remarkably, we showed that histamine significantly reverted both CREB and PSD-95 impairments induced by LPS. In agreement with our previous experiments, a differential response to histamine was observed according to the inflammatory context. Histamine *per se* caused a slightly increase trend of hippocampal inflammation and neurogenesis. However, when preceded by a peripheral inflammatory stimulus, histamine led to reversion of neuroinflammation, neurogenesis impairments and cognitive deficits. These results indicate that histamine is able to revert LPS-induced deficits in the mouse DG, suggesting a possible contribution of histamine in terms of cognition improvement in a neuroinflammatory context, as well as a possible application of this molecule in the treatment of neurological disorders, such as AD, PD and stroke. Therefore, future studies should be done to unveil possible behavior and electrophysiological changes associated to histamine treatment after LPS peripheral administration. Moreover, it would be very interesting to assess more timepoints for histamine administration in order to establish a therapeutic interval where histamine could be used as a therapy against generalized infections.

## Conclusion

Histamine has been suggested as an important modulator of several CNS functions. Cumulative data have demonstrated a dual role of histamine under different environmental contexts (physiological vs pathological), probably due to the triggering of different receptors and signaling pathways. Histamine *per se* induces a microglial pro-inflammatory phenotype, while it has protective effects under an inflammatory challenge mimicked by LPS counteracting microglial responses. To the best of our knowledge, this is the first study showing the dual effect of histamine on neuroinflammation and neurogenesis in the mouse hippocampus *in vivo*. Herein, we demonstrated that histamine is able to revert LPS-induced hippocampal neuroinflammation by reducing not only the expression levels of markers for activated glial cells but also by decreasing the production of pro-inflammatory molecules. Additionally, histamine was able to increase, at long-term, the survival of newborn cells in the DG niche of LPS-challenged mice as well as to counteract the SGZ neuroblast loss of volume and dendritic arbor complexity. Histamine was able to inhibit LPS-induced decrease on markers correlated with neuronal functionality and synaptic strength, indicating a reversion of LPS-induced cognitive decline. Interestingly, the protective actions of histamine were stronger when a higher concentration of LPS was used, possibly indicating a dose-dependent effect of LPS in the CNS inflammatory response. This could be a result of differential immune memory triggered by the two LPS dosages used (training vs tolerance) that consequently might have promoted a different response to the second inflammatory stimulus (histamine).

Strategies involving attenuation or blockage of the brain inflammatory response in conjugation with the amelioration of neurogenesis impairments might represent a valid therapeutic strategy for several neurodegenerative pathologies. Therefore, our results highlight the potential of histamine as a promising therapeutic agent for conditions that involve neuroinflammation and cognitive deficits.

## Supplementary information

Supplementary data

## Data Availability

The datasets generated during and/or analyzed during the current study are available from the corresponding author on reasonable request.
