# Supplementary material for: Histamine modulates hippocampal inflammation and neurogenesis in adult mice
Source: Sci Rep. 2019 Jun 10;9:8384. doi: 10.1038/s41598-019-44816-w (PMC6558030; doi:10.1038/s41598-019-44816-w)
Supplement: Supplementary file 1 — Supplementary data [file 41598_2019_44816_MOESM1_ESM.pdf]

Histamine modulates hippocampal inflammation and neurogenesis in adult mice

Cláudia Saraiva[1+], Sandra Barata-Antunes[1+], Tiago Santos[1], Elisabete Ferreiro[2], Ana Clara Cristóvão[1], Catarina Serra-Almeida[1], Raquel Ferreira[1], Liliana Bernardino[1*]

1- CICS-UBI - Health Sciences Research Centre, University of Beira Interior, 6201-001Covilhã, Portugal; 2- Center for Neuroscience and Cell Biology, 3004-504 Coimbra, Portugal;
+ Both authors contributed equally
* Corresponding authors: libernardino@fcsaude.ubi.pt

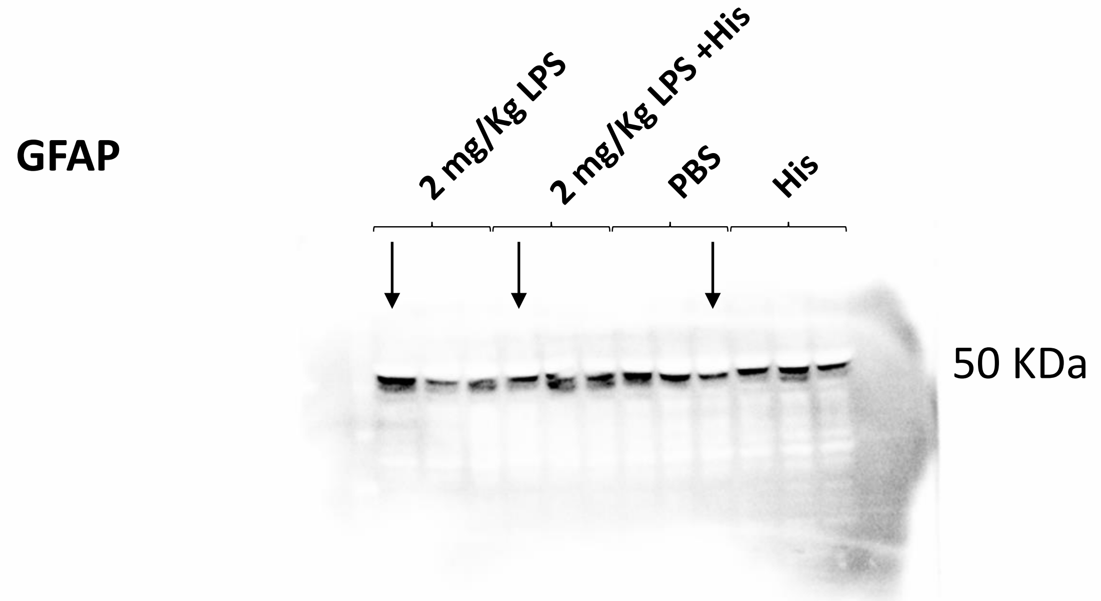

↓ = Selected bands for representative images

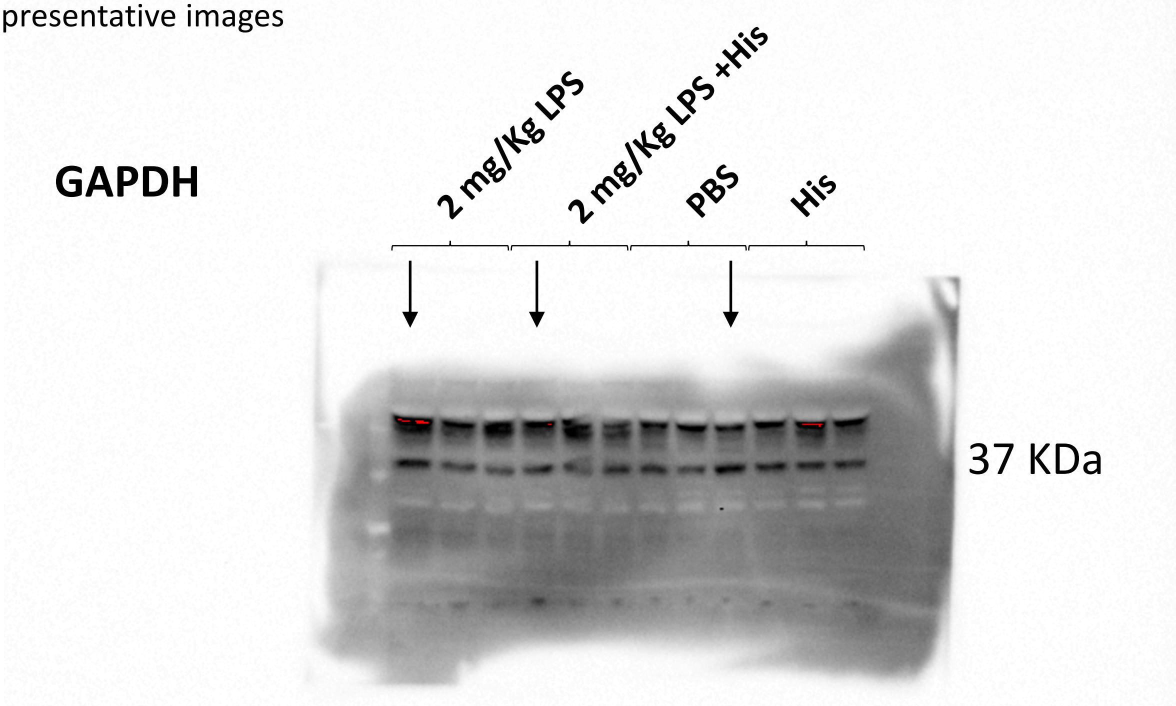

Supplementary Figure 1. Full-length membranes used for quantification of GAFP. Protein immunoreactive bands were visualized in a Chemidoc™MP imaging system (BioRad Laboratories) after incubation with NZY supreme ECL reagent (NZYTech, Lisbon, Portugal). Densitometric analysis was performed using the software ImageLab (BioRad Laboratories).

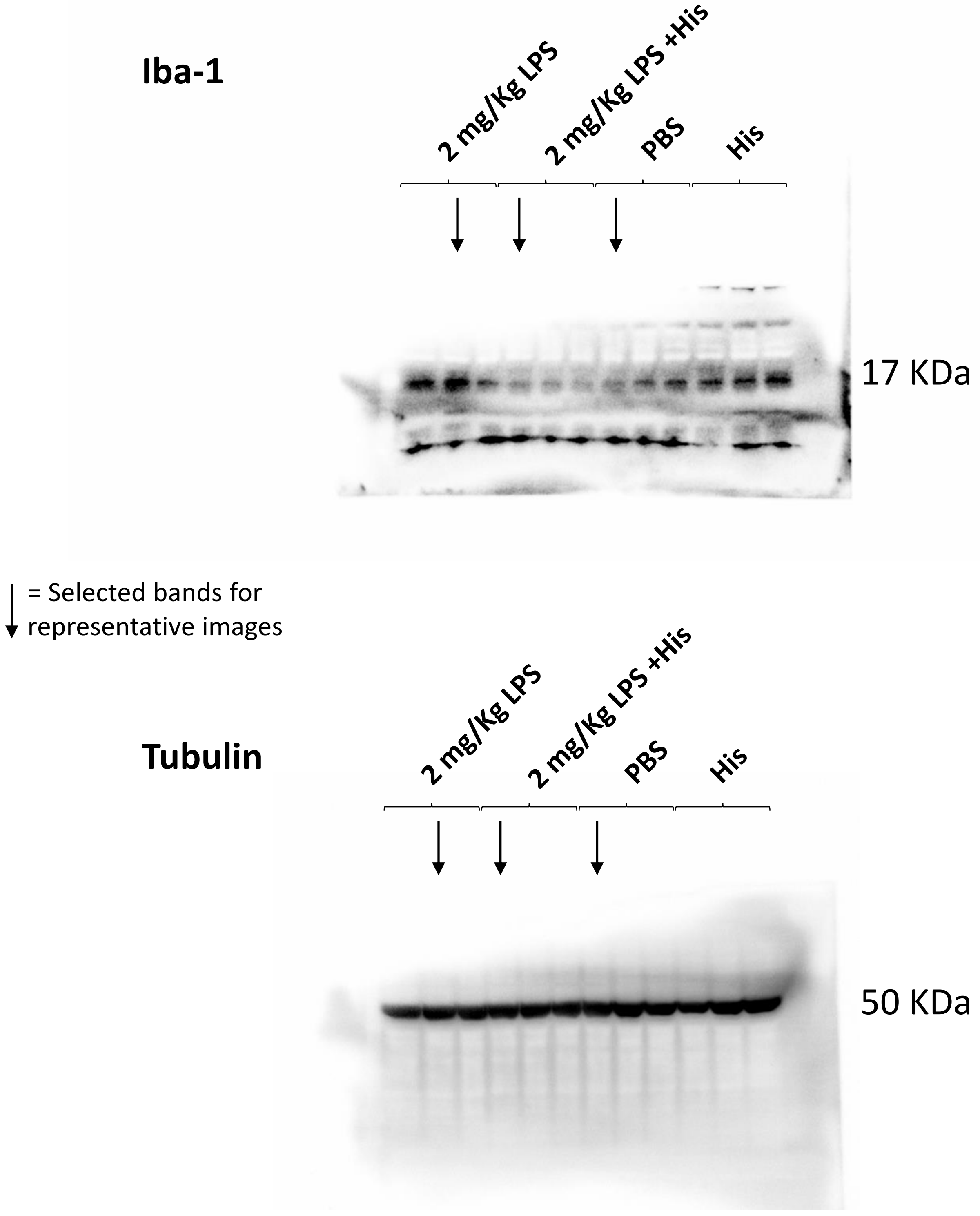

Supplementary Figure 2. Full-length membranes used for quantification of Iba-1. Protein immunoreactive bands were visualized in a Chemidoc^{TM}MP imaging system (BioRad Laboratories) after incubation with NZY supreme ECL reagent (NZYTech, Lisbon, Portugal). Densitometric analysis was performed using the software ImageLab (BioRad Laboratories).

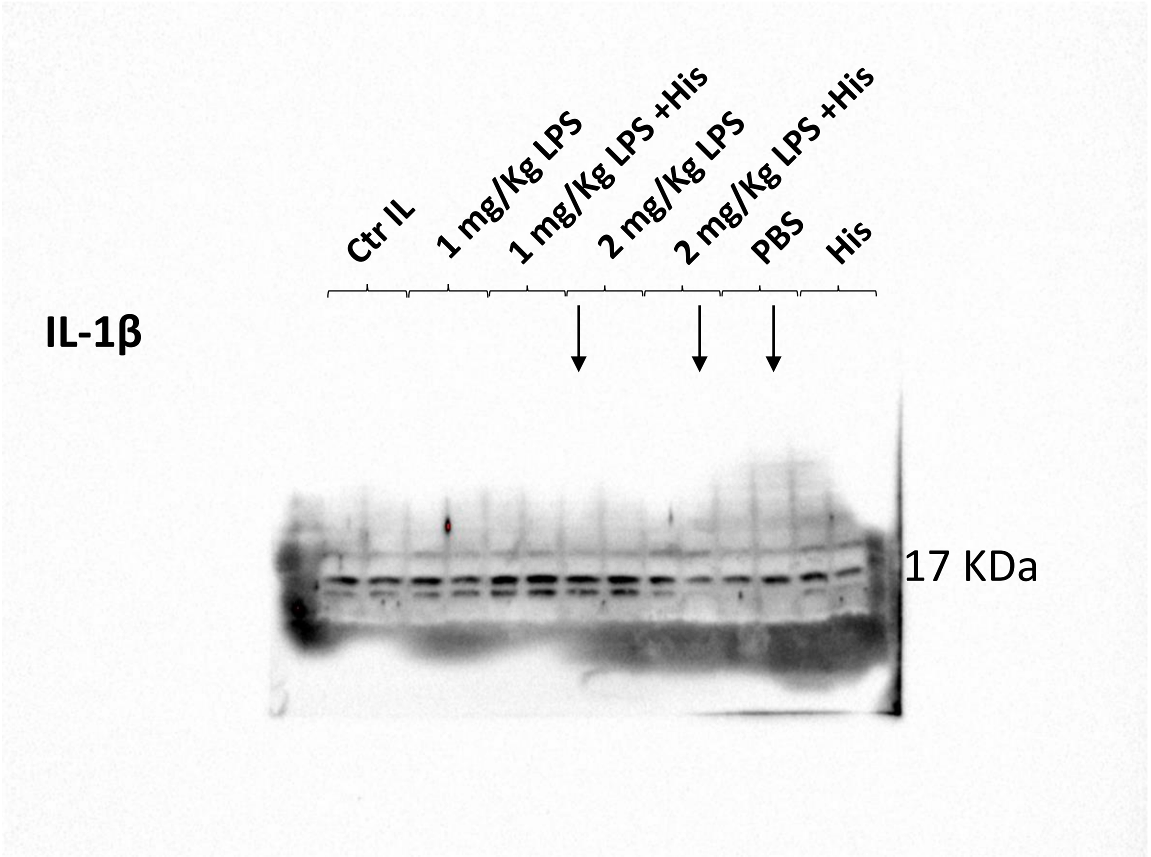

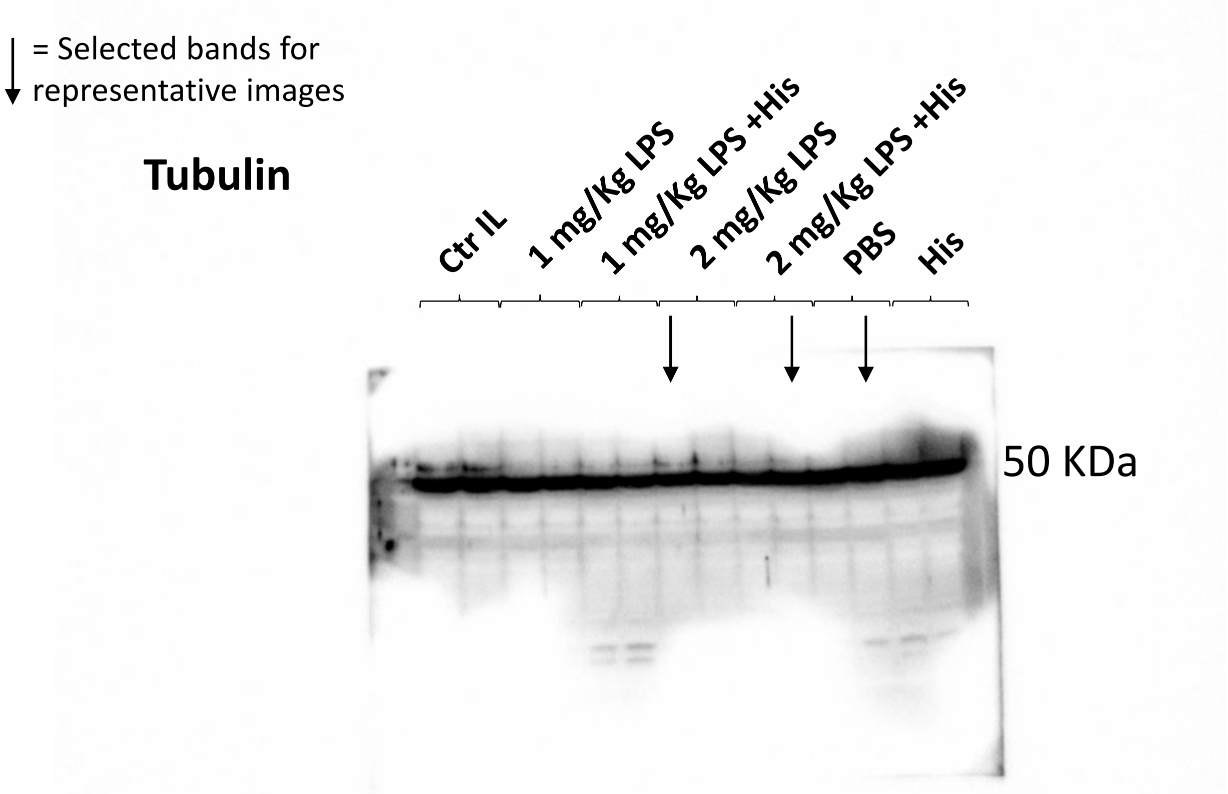

Supplementary Figure 3. Full-length membranes used for quantification of IL1-β. Protein immunoreactive bands were visualized in a Chemidoc™MP imaging system (BioRad Laboratories) after incubation with NZY supreme ECL reagent (NZYTech, Lisbon, Portugal). Densitometric analysis was performed using the software ImageLab (BioRad Laboratories).

Ctrl IL - Ipsilateral hemisphere of mice subjected to i.p. injections of PBS and to intracerebral injection of PBS.

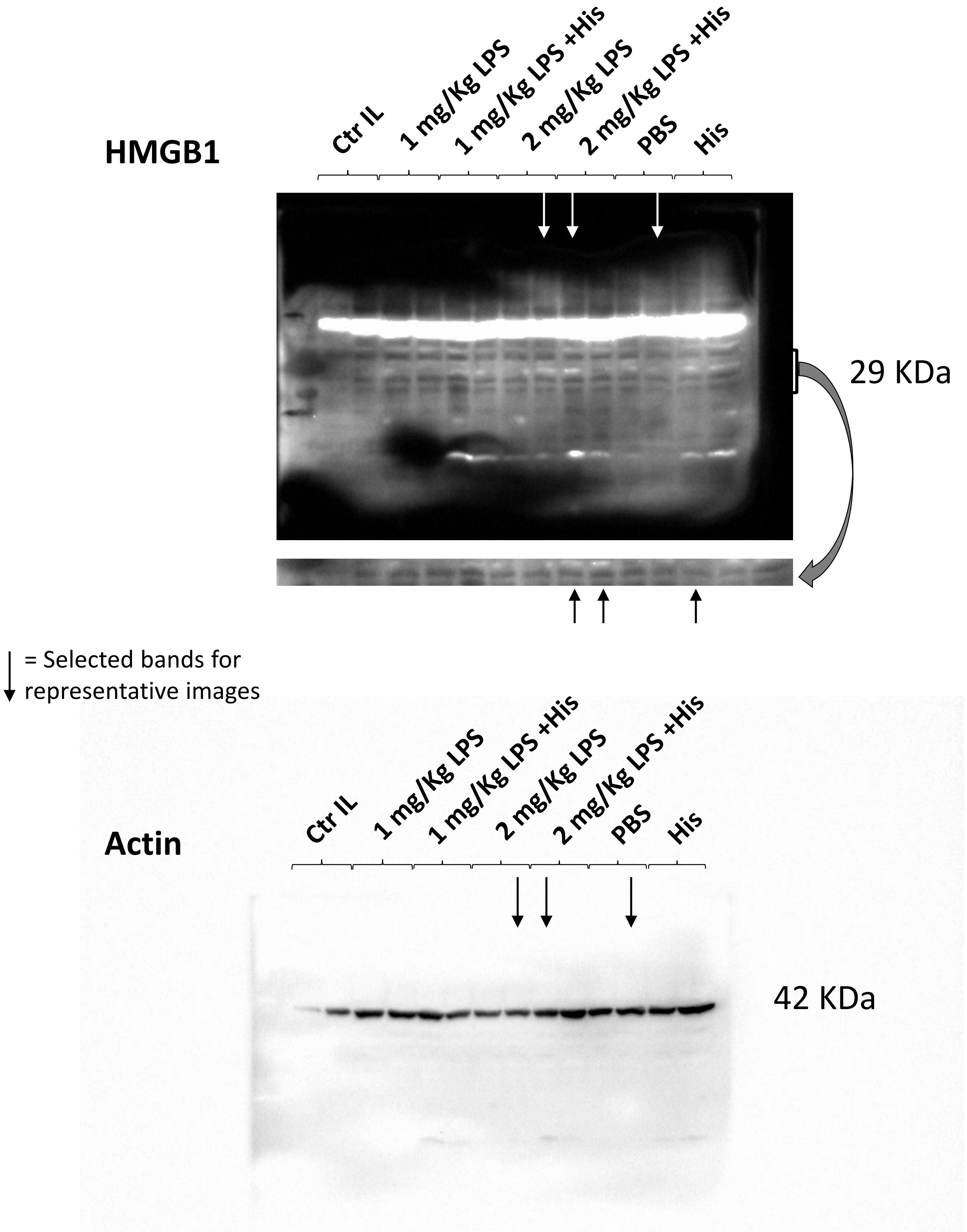

Supplementary Figure 4. Full-length membranes used for quantification of HMGB1. Protein immunoreactive bands were visualized in a Chemidoc^{TM}MP imaging system (BioRad Laboratories) after incubation with NZY supreme ECL reagent (NZYTech, Lisbon, Portugal). Densitometric analysis was performed using the software ImageLab (BioRad Laboratories).

Ctrl IL - Ipsilateral hemisphere of mice subjected to i.p. injections of PBS and to intracerebral injection of PBS.

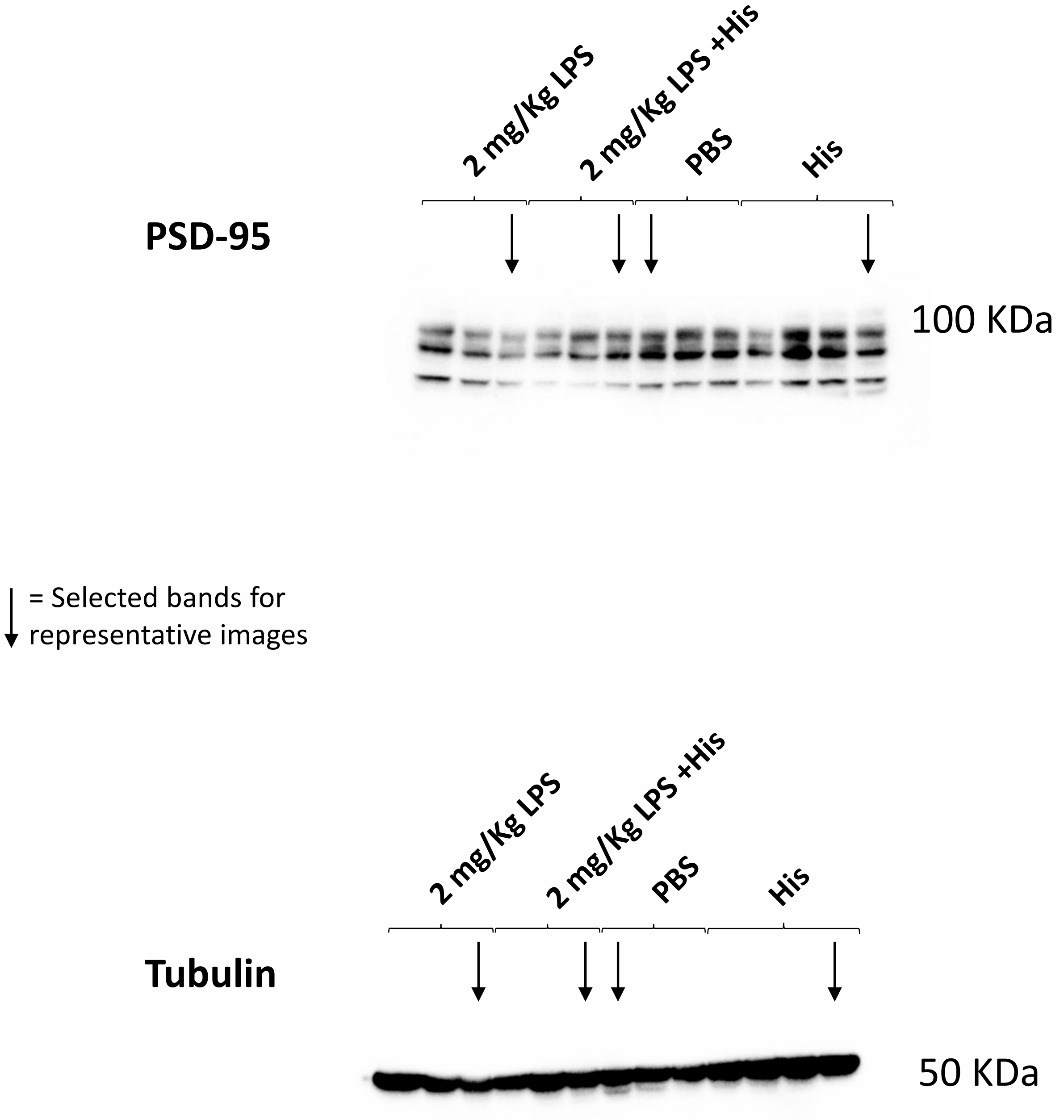

Supplementary Figure 5. Full-length membranes used for quantification of PSD-95. Protein immunoreactive bands were visualized in a Chemidoc™MP imaging system (BioRad Laboratories) after incubation with NZY supreme ECL reagent (NZYTech, Lisbon, Portugal). Densitometric analysis was performed using the software ImageLab (BioRad Laboratories).

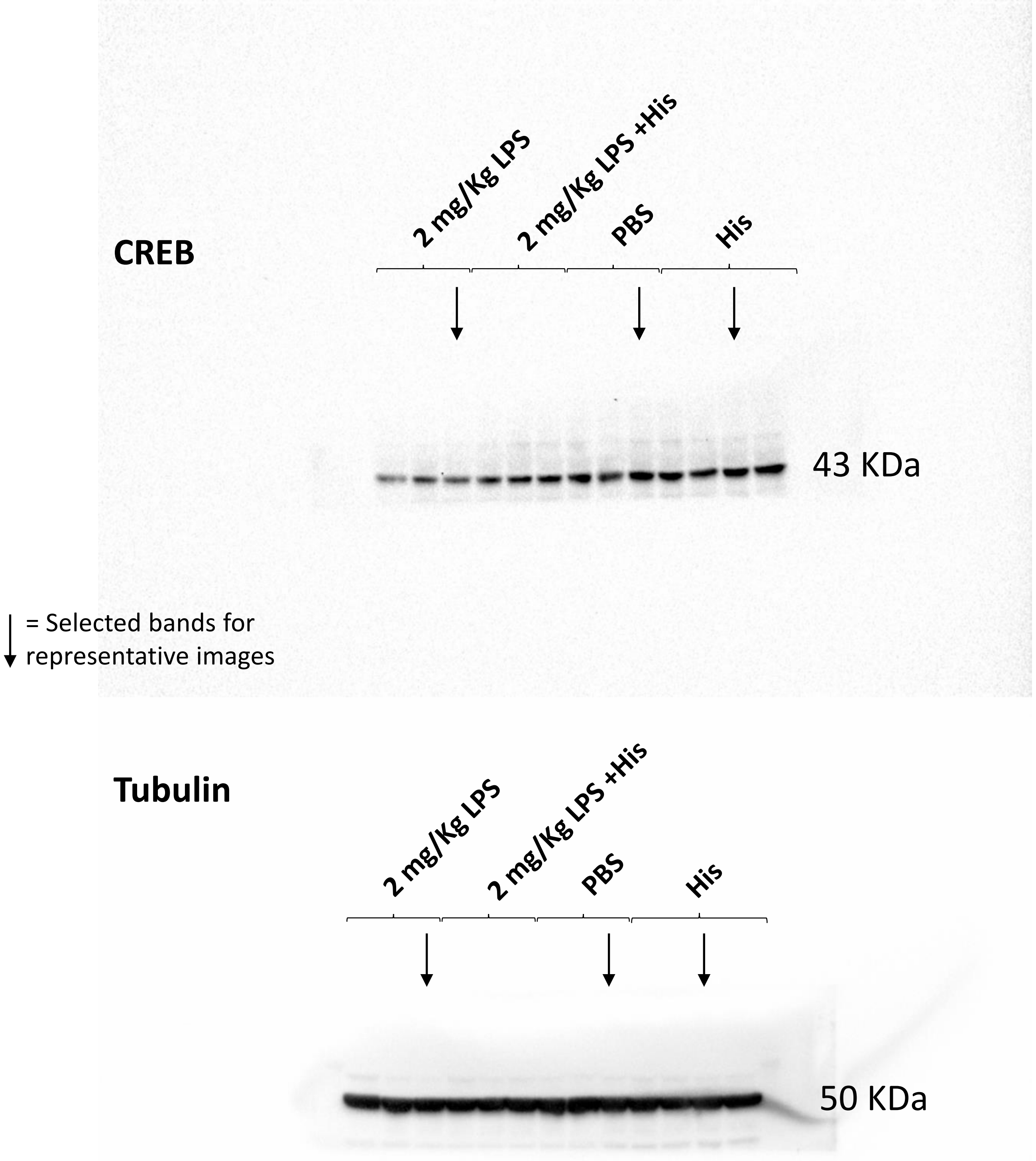

Supplementary Figure 6. Full-length membranes used for quantification of CREB. Protein immunoreactive bands were visualized in a Chemidoc™MP imaging system (BioRad Laboratories) after incubation with NZY supreme ECL reagent (NZYTech, Lisbon, Portugal). Densitometric analysis was performed using the software ImageLab (BioRad Laboratories).

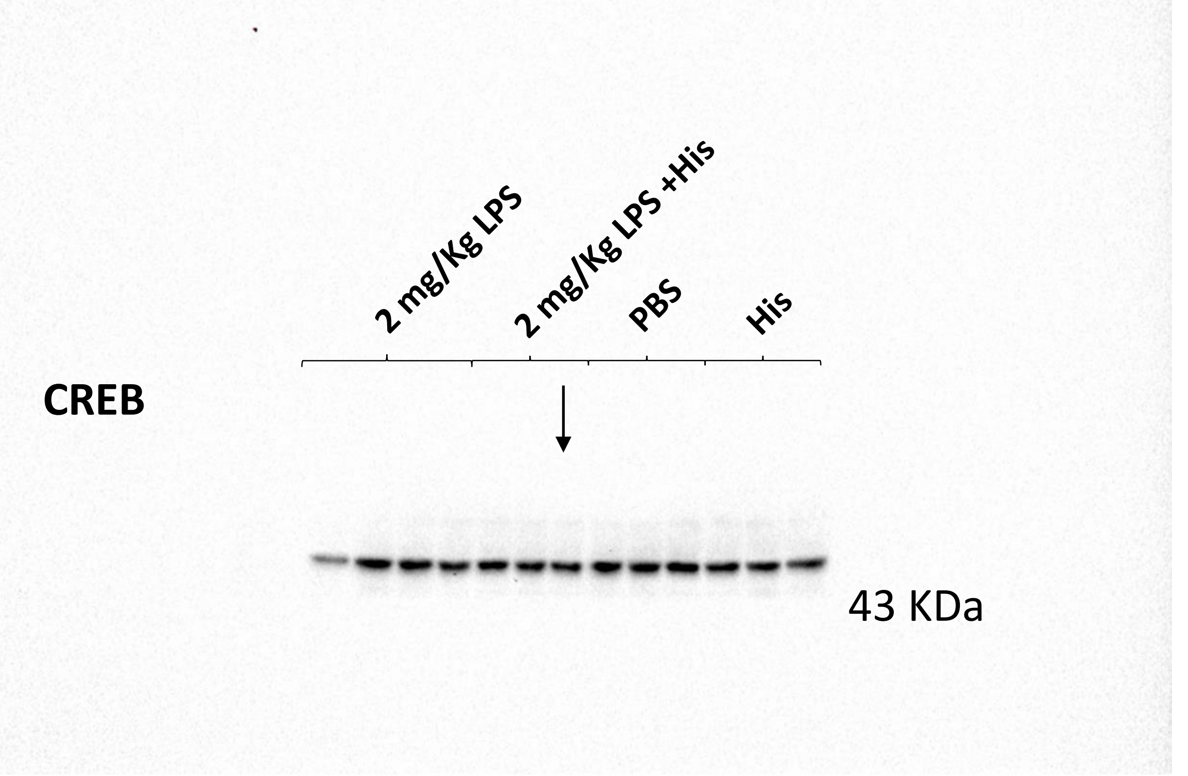

= Selected bands for representative images

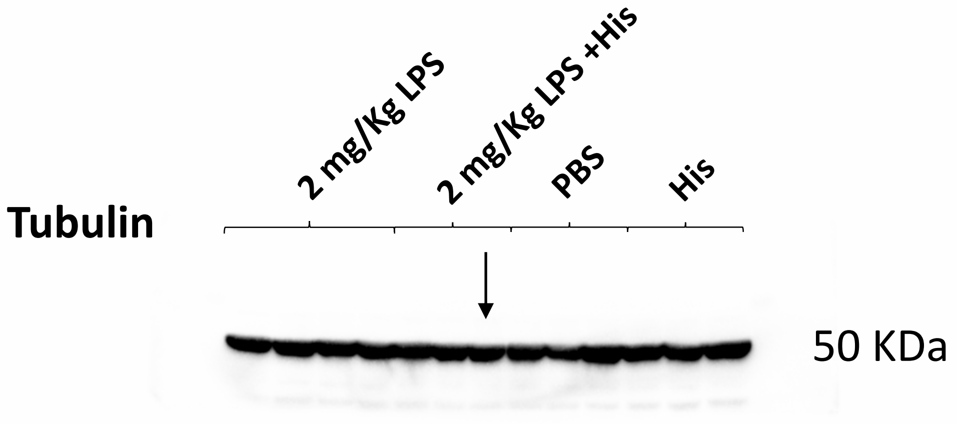

Supplementary Figure 7. Full-length membranes used for quantification of CREB. Protein immunoreactive bands were visualized in a Chemidoc^{TM}MP imaging system (BioRad Laboratories) after incubation with NZY supreme ECL reagent (NZYTech, Lisbon, Portugal). Densitometric analysis was performed using the software ImageLab (BioRad Laboratories).
